# Neutralizing Autoantibodies Against IL-10 in Inflammatory Bowel Disease

**DOI:** 10.1056/NEJMoa2312302

**Published:** 2024-08-01

**Authors:** Helen Griffin, Lourdes Ceron-Gutierrez, Nima Gharahdaghi, Soraya Ebrahimi, Sophie Davies, Peh Sun Loo, Andras Szabo, Eleri Williams, Anirban Mukhopadhyay, Louise McLoughlin, Steven Irwin, Simon Travis, Paul Klenerman, Su Bunn, Andrew J. Cant, Sophie Hambleton, Holm H. Uhlig, Rainer Doffinger

**Affiliations:** 1 Immunity & Inflammation Theme, Newcastle University Translational and Clinical Research Institute, Newcastle upon Tyne, UK; 2 Department of Clinical Biochemistry and Immunology, Cambridge University Hospital, Cambridge, UK; 3 Translational Gastroenterology Unit, University of Oxford, Oxford, UK; 4 Great North Children’s Hospital, Newcastle upon Tyne Hospitals NHS Foundation Trust, Newcastle upon Tyne, UK; 5 Paediatric Gastroenterology Department, Heim Pal National Paediatric Institute, Budapest, Hungary; 6 Dept of Paediatric Gastroenterology, Royal Belfast Hospital for Sick Children, Belfast, UK; 7 Dept of Pathology, Royal Victoria Hospital, Belfast Health and Social Care Trust, Belfast, UK; 8 Kennedy Institute of Rheumatology, University of Oxford, Oxford, UK; 9 NIHR Biomedical Research Centre, University of Oxford, Oxford, UK; 10 Department of Paediatric Gastroenterology, Royal Aberdeen Children’s Hospital, Aberdeen, UK; 11 Department of Paediatrics, University of Oxford, Oxford, UK; 12 National Institute for Health Research (NIHR), Cambridge, UK

## Abstract

We discovered high titer neutralizing autoantibodies against interleukin-10 (anti-IL-10) in a child with infantile onset IBD, phenocopying inborn errors of IL-10 signalling. Following B cell depletion and associated decline in the anti-IL-10 titer, conventional IBD therapy could be withdrawn. A second child with neutralizing antibodies against IL-10 displayed a milder course of IBD and has been managed without B cell depletion. We conclude that neutralizing autoantibodies against IL-10 may be a causative or modifying factor in IBD, with potential implications for therapy.

## Introduction

Interleukin-10 (IL-10) serves as an anti-inflammatory cytokine with a crucial role in controlling intestinal inflammation. Biallelic loss-of-function variants of IL-10 or its receptor subunits (encoded by *IL10, IL10RA* and *IL10RB* respectively) lead to severe, infantile onset, ulcerating intestinal inflammation with fistulizing disease^1,2^. This condition presents with infantile onset enterocolitis in most patients, and some may manifest additional features such as folliculitis, arthritis, and B cell lymphoma^1,2^. This phenotype and the curative potential of hematopoietic stem cell transplantation in such diseases highlight the significance of IL-10 signalling in immune cells as an immunoregulatory mechanism. Interestingly, a missense variant in *IL10RA* increases the risk of adult onset polygenic inflammatory bowel disease (IBD)^3^, while regulatory variants that boost IL-10 expression are associated with reduced IBD susceptibility.^4^ These data suggest an impact of IL-10 signalling in both early-onset and adult IBD but it remains possible that the absence of IL-10 signaling in infancy has a more profound effect on gut immune homeostasis because of a developmental window of susceptibility.

The field of inborn errors of immunity (IEI) encompasses a wide array of genetic disorders, some of which can be mimicked by autoantibodies targeting the same pathways (“phenocopies” of IEI)^5,6^. Among these, neutralizing autoantibodies against interferon-gamma (IFN-γ), IL-23, type I interferon, GM-CSF and IL-6 have each been associated with phenotypes similar to biallelic loss of function variants of their respective receptors^5, 6,7^. While autoantibodies against GM-CSF have long been recognised in individuals with Crohn’s disease^8^, this is by no means a phenocopy of the pulmonary alveolar proteinosis associated with either genetic deficiency of the GM-CSF receptor^9^ or isolated acquisition of neutralizing autoantibodies to GM-CSF^10^ and tends to imply a bystander rather than a pathogenic role for anti-GM-CSF autoantibodies in IBD.

In this context, our study identifies elevated titers of neutralizing autoantibodies against IL-10 in two patients with very early onset IBD.

## Case Reports

### Clinical Presentation and Evaluation of index patient 1

**Figure 1:**
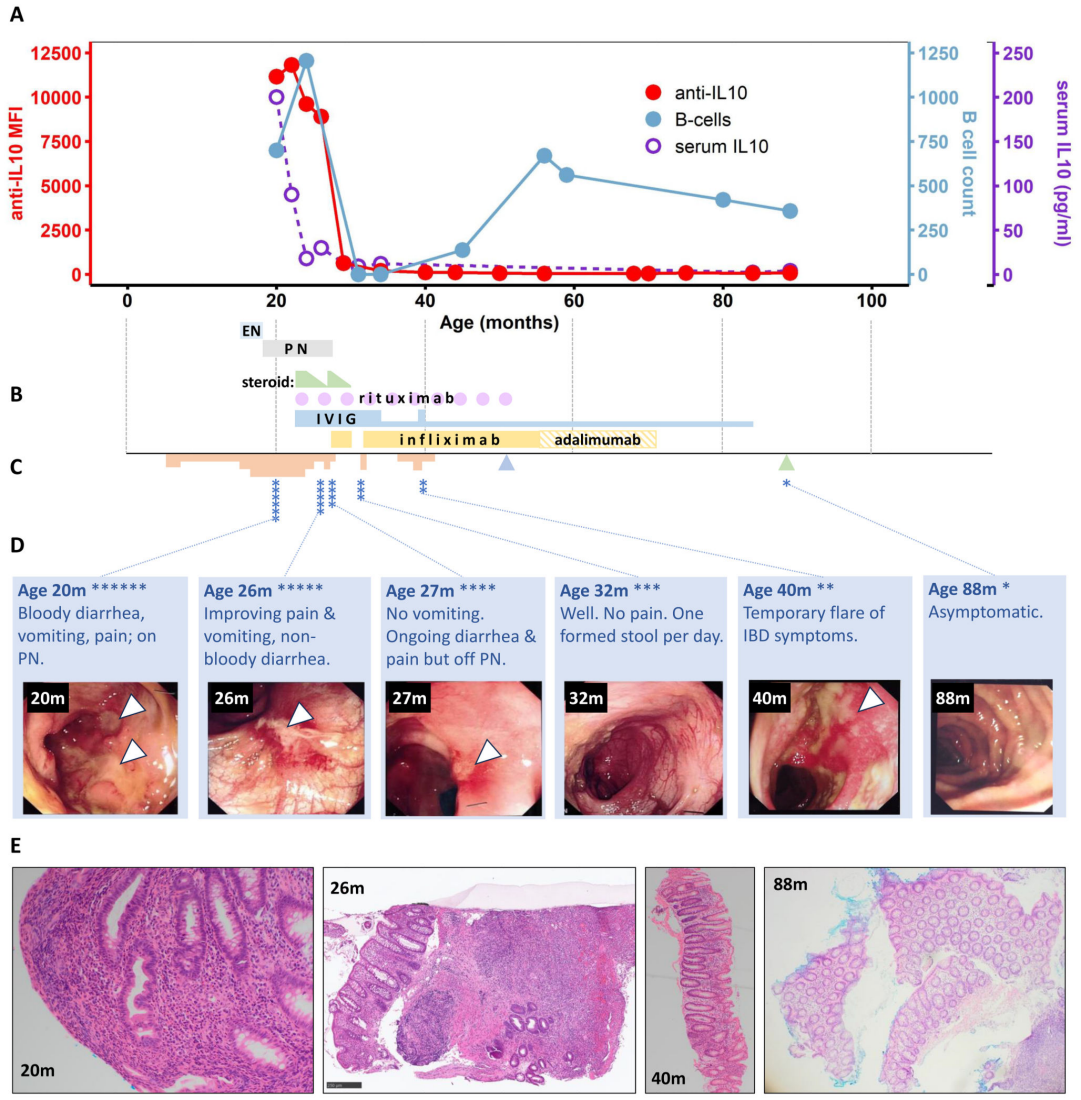
Very early onset inflammatory bowel disease (VEOIBD) in P1, a child with high titer neutralizing autoantibodies to interleukin-10. (A) Serum anti-IL-10 titer (red, Mean Fluorescence Intensity (MFI) by particle-based assay, 1:100 dilution), IL-10 (purple) and B cell number (blue) over time while receiving therapy as indicated in (B); EN, exclusive enteral nutrition; PN, parenteral nutrition; IVIG, intravenous immunoglobulin (high dose/replacement dose indicated by height of bar). (C) Clinical timeline in which the severity of GI symptoms is indicated by the height of the pink shading. Asterisks show the timing of endoscopic examinations. Triangles at 52 (blue) and 84 (green) months represent brief episodes of oedema and altered fecal calprotectin, respectively, as described in the text. (D) Sequential colonoscopic appearances showing improvement in ulceration (arrows) over time, left to right. (E) Representative images of colonic histopathology at indicated time-points, stained with hematoxylin and eosin. Left: mild distortion of glandular architecture associated with increased cellularity of the lamina propria and admixed acute and chronic inflammation at 20-26 m of age. Right: progressive resolution of inflammatory changes & restoration of normal architecture.

A previously well female Caucasian infant, P1, developed bloody diarrhea and vomiting from the age of 3 months. There was no family history of IBD or IEI. Dietary restrictions including dairy exclusion initially produced partial improvement but a severe deterioration in symptoms with accompanying weight loss from 17 months of age was refractory to exclusive enteral feeding with an elemental diet (see clinical timeline in Figure 1B,C). Endoscopic examination revealed moderate gastritis and severe pancolitis with deep, rolled-edged ulcers, equating to A1a L2 L4a B1 G1 by the Paris classification^11^; this was associated with acute inflammatory histopathological features but not granulomata (Figure 1D,E). Parallel microbiological investigations revealed toxin-producing *Clostridium difficile* on a single occasion but there was no change in symptoms upon treatment with metronidazole or vancomycin. The child required multiple transfusions, total parenteral nutrition and gut rest and was referred to a tertiary service for evaluation and management with a suspicion of monogenic IBD.

**Figure 2:**
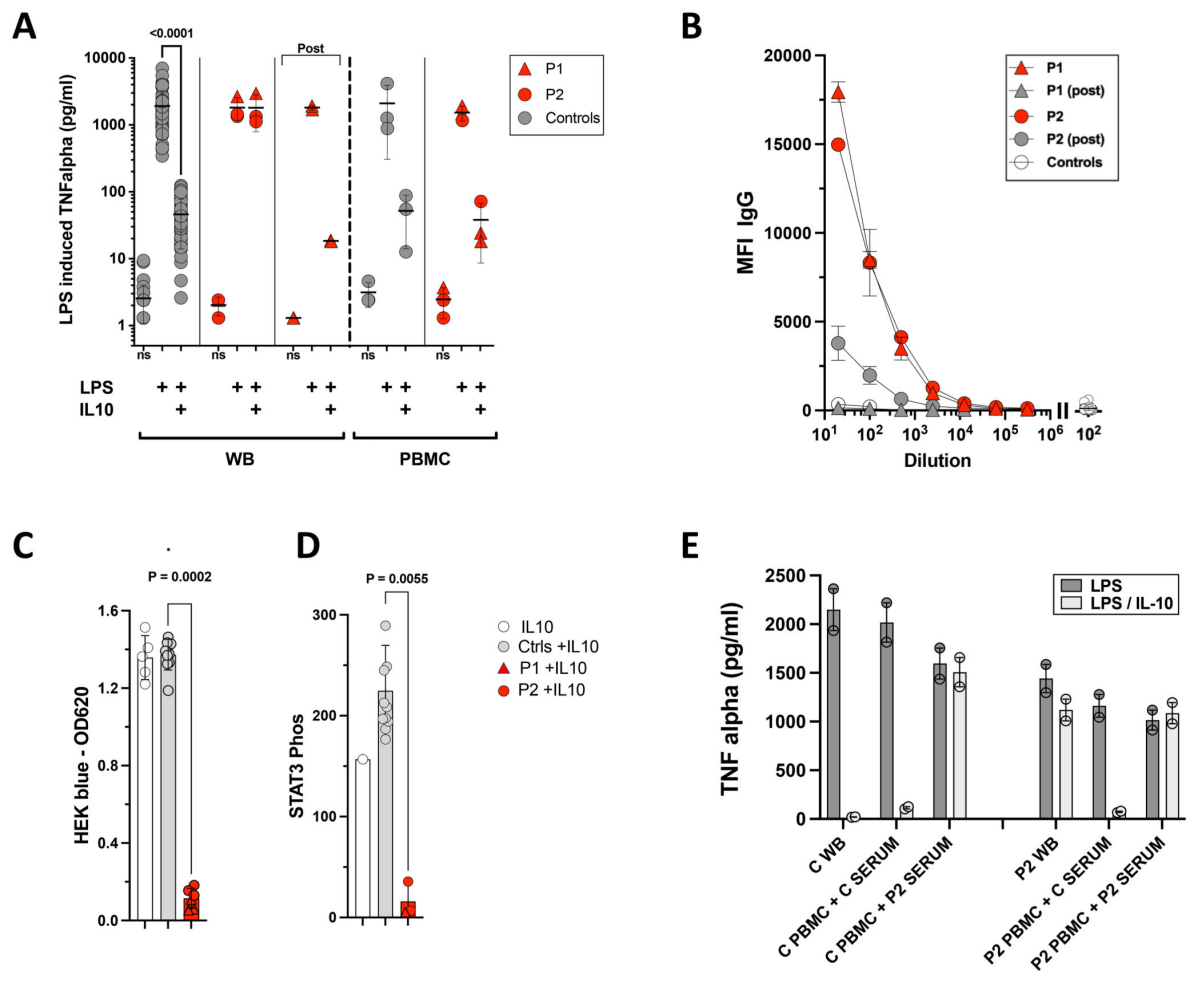
Identification and functional characterization of autoantibodies to interleukin-10 in 2 children with VEOIBD. **A: IL-10-mediated downregulation of LPS induced TNF alpha in Whole Blood (WB) and PBMC**. Whole blood and PBMCs of healthy controls (WB n=50, PBMC n = 3) and patients (P1, P2) were activated with lipopolysaccharide (LPS) alone or in the presence of IL-10. Addition of LPS induces strong up-regulation of TNF alpha production when compared to non-stimulated samples (ns). Control WB and PBMC show significant downregulation of LPS-induced TNFα production when co-stimulated with IL-10, while there is no downregulation in WB of P1 and P2. Patient responses are restored when stimulating PBMC in the absence of autologous serum. Patient P1’s WB was also tested after treatment, when anti-IL-10 had declined (“Post”), showing a normal response similar to controls. Mean values with standard deviations (SD) are shown. IL10-mediated downregulation of WB controls was analysed by Mann-Whitney test. **B: Titration of anti-IL-10 IgG**. Sera of P1 and P2 were tested by particle-based flow cytometry for the presence of auto-antibodies to IL-10. Titrations for anti-IL-10 IgG were performed in 1/5 dilution steps starting at a dilution of 1/20. Titers at initial presentation and post treatment are shown for P1 and P2 (mean values +/- SD from duplicate testing). 20 healthy adult controls (mean +/- SD) are as well shown at dilutions 1/20 and 1/100. Results from an additional cohort of 50 healthy pediatric control sera at a dilution of 1/100 are shown on the right side. **C: IL-10 reporter assay (HEK-blue™ IL-10 cells)**. An IL-10 responder line was activated with IL-10 either alone (4 ng/ml, n=5, white bars/circles) or in the presence of control serum (gray bars/circles, n=10) or patient serum P1 (red bars/triangles, 1:10, n=3) or P2 (red bars/circles, 1:10, n=3). Sera of P1 and P2 significantly inhibit IL-10-induced SEAP induction (Mann-Whitney, p < 0.0002) as determined by colorimetric measurement at OD620. **D: IL-10-induced STAT3 activation in a dual luciferase reporter assay**. Effect of patient or control serum on IL-10-mediated STAT3 transcriptional activity. Sera were tested for their ability to interfere with IL-10 induced STAT3 activation of a reporter-transfected cell line, measured by luciferase induction (Mann-Whitney, p < 0.0055). Activation was performed either with IL-10 alone (200 pg/ml), or in the presence of control serum (gray bar/circles, n=10) or patient serum of P1 (red bar/triangle, 1:10) or P2 (red bars/circles, 1:10). **E: IL-10 cross-inhibition assay**. PBMC of patient P2 and a healthy control (C) were activated by LPS alone or LPS + IL-10, in the presence of either control serum or P2 serum. Comparison is made with responses in whole blood (WB). Bars indicated mean values of 2 replicates from 1 experiment, symbols show individual values. P2 serum blocks IL-10-mediated downregulation of TNF alpha by control and patient cells.

P1 underwent an extensive diagnostic evaluation, revealing a normal immune profile (neutrophil oxidative burst, lymphocyte subsets, total and vaccine-specific immunoglobulins) and a negative screen for pathogenic variants in genes associated with monogenic IBD or IEI^5^ by both targeted panel and whole exome sequencing. Since cryptic variants in IL-10 receptor genes have been described, we performed a functional assay of the ability of exogenous IL-10 to suppress LPS-stimulated production of tumor necrosis factor (TNF) by patient cells^1,12,13^. A whole blood assay indicated unresponsiveness of P1’s cells to IL-10 but, upon isolation by density gradient centrifugation and thorough washing, patient peripheral blood mononuclear cells (PBMC) recovered a normal response (Figure 2A). This suggested integrity of the IL-10 receptor signalling pathway and the presence of a serum factor that negatively interfered with IL-10 signalling. P1 serum was therefore analysed for the presence of auto-antibodies to IL-10, revealing a high titer (Figures 1A, 2B) along with highly elevated serum concentrations of IL-10 (200 pg/mL; Figure 1A). The capacity of these autoantibodies to neutralize IL-10 functionally was confirmed by the ability of P1 serum to suppress IL-10 signalling in whole blood (Figure 2A) and as well by two independent IL-10 reporter assays (Figure 2C,D). An extensive serologic screen was negative for autoantibodies against other cytokines (IL-1 beta, IL-6, IL-8, IL-12, IL-23, IL-17A, IL-17F, IL-21, IL-22, IL-27, G-CSF, GM-CSF, TNF alpha), interferons (IFN alpha, IFN beta, IFN omega, IFN Lambda 1, IFN Lambda 2, IFN Lambda 3, IFN gamma) or autoantigens implicated in rheumatological or autoimmune diseases (a broad panel including nuclear, adrenal, islet, ovarian, parathyroid and enterocyte antigens).

The family were extensively counselled about the urgent need for immunomodulatory therapy. Given the severe clinical phenotype and the predicted pathogenic effect of the high titer autoantibodies to IL-10, conventional treatment with intravenous corticosteroid was augmented with high dose intravenous immunoglobulin (IVIG 2g·kg^-1^ fortnightly) and B cell depleting therapy (rituximab 1.5 g·m^-2^ in divided doses, 3-monthly). This combined approach led to a prompt response, both clinically and histologically (Figure 1D,E). Symptoms began to recur upon corticosteroid wean and infliximab was therefore added successfully as a steroid-sparing agent. When serum anti-IL-10 titers finally declined to undetectable levels (from 31 months onwards), infliximab was initially withdrawn, coinciding with a relapse in symptoms which resolved when it was restarted. Due to the severity of her presenting disease, the patient therefore remained on combination therapy for a further 2-year period, during which immunoglobulin was switched to replacement therapy (~ 0.5 g/kg/month) that continued along with anti-TNF. The patient had a relapse of IBD symptoms at 40 months of age managed with high dose IVIG, and one episode of self-resolving unexplained generalised oedema at 52 months requiring intensive care. Due to difficulties with intravenous access, infliximab was switched to adalimumab at 56 months, and all immunomodulatory therapy withdrawn at 72 months of age. A year later, laboratory markers including transiently raised fecal calprotectin (1400µg/g) produced concern for relapse but the patient remained clinically asymptomatic and upper and lower GI endoscopy revealed normal appearances (Figure 1D); markers subsequently normalized spontaneously. The patient remains well at almost 10 years of age with normal bowel habit and fecal calprotectin (37 µg/g), but is hypogammaglobulinemic, despite the reappearance of B cells (Figures 1A, S1). Anti-IL-10 autoantibodies remain fully suppressed.

### Clinical Presentation and Evaluation of Patient 2

An unrelated female child (P2) with past history of eczema and recurrent urinary tract infections, developed bloody diarrhea and abdominal pain with rising fecal calprotectin (peaking at >8000µg/g) from the age of 4.5 years. The family history was negative for IBD. Endoscopic examination revealed a moderately active pancolitis with aphthous ulceration (i.e., rectum to mid-transverse colon and cecum; PARIS classification A1a L2/L4a B1 G0^11^) (Figure S2D). There was no perianal disease and the upper gastrointestinal tract appeared normal, except for mild chronic gastritis. Histologically, colorectal biopsies showed mild crypt architectural distortion from cecum to rectum with Paneth cell metaplasia indicating features of chronic mucosal damage (Figure S2E). There were acute and chronic inflammatory cell infiltrates in the lamina propria, with loss of normal gradients and some patchy basal plasmacytosis. There was acute cryptitis, with intraepithelial neutrophils and mucin-depletion. The appearances were of mild to moderate chronic active inflammation from cecum to rectum, with a seemingly continuous but slightly patchy distribution, without granulomata or viral inclusions. These features supported the diagnosis of IBD unclassified.

A targeted clinical genetic screen revealed no pathogenic variants in genes linked to monogenic IBD or IEI and standard immunophenotyping was normal. A whole blood assay, similar to the one performed in P1, showed the same failure of IL-10 to downregulate LPS-induced production of TNF (Figure 2A). Whereas anti-IL-10 autoantibodies were present in high titer (Figure S2A, 2B), IL-10 could not be detected in patient plasma, after specific *in vitro* activation nor when spiked with exogenous IL-10, implying competition of the detection reagent with an endogenous factor. Consistent with neutralizing activity of the autoantibodies to IL-10, P2 serum prevented signal transduction upon exposure to IL-10 in two independent reporter assays (Figure 2C, D). In a cross-inhibition assay, P2 serum prevented the IL-10-dependent downregulation of the TNFα response to LPS of both autologous and control PBMC (Figure 2E). No other anti-cytokine autoantibodies could be detected.

Due to the less severe presentation compared to P1, P2 was managed without B cell-depleting therapy (Figure S2B,C). Initial management with steroids showed a positive response, but remission could not be sustained with azathioprine as steroids were weaned. Infliximab was therefore added as a steroid-sparing agent, and azathioprine was later withdrawn. Over the following 2 years, the child has mostly been in remission but with occasional flares of bloody diarrhea and abdominal pain during which fecal calprotectin is raised. The titer of neutralizing anti-IL10 has reduced, but not disappeared (Figure 2B, S2A).

## Discussion

High titer neutralizing anti-IL-10 antibodies were identified in a child with infantile-onset severe colitis that phenotypically resembled an inborn error of the IL-10 pathway, despite the absence of pathogenic mutations. This finding aligns with mouse studies demonstrating the potential of anti-IL-10 antibodies to induce or aggravate colitis *in vivo*^14^ and prior reports of anti-IL-10 autoantibodies in various immune-mediated disorders. The latter included small numbers of individuals with IBD^15,16^, albeit none amounting to a phenocopy or treated with B cell-directed therapy, and with variable neutralizing capacity (summarized in supplementary Table S1). To target autoantibody production in our patient, we combined anti-B cell therapy (rituximab) with gut-directed immunosuppression. Following the disappearance of anti-IL-10, stable resolution of clinical symptoms and gastrointestinal pathology was ultimately achieved. The likely pathogenic role of the autoantibodies is supported by the exceptionally close phenocopy with monogenic disorders of the IL-10 pathway, their neutralizing capacity *in vitro* and the brisk therapeutic response to anti-CD20 B cell targeting therapy alongside more conventional anti-inflammatory treatment. This links a specific plausible pro-inflammatory mechanism with supportive evidence *in vitro* and a therapeutic intervention.

Subsequently, neutralizing anti-IL10 autoantibodies were identified in an older child with milder colitis, suggesting a potential spectrum of disease and/or a disease-modifying effect. Both patients exhibited markedly elevated titers of neutralizing anti-IL-10 antibodies (fig 2B) but IL-10 is not the only source of regulatory restraint on GI inflammation. Existing genetic and animal studies have indicated that near-complete blockade of IL-10 signaling is necessary to induce intestinal inflammation. Even complete deficiency of the IL-10 pathway through biallelic loss-of-function mutations produces a spectrum of disease, suggesting a nuanced interplay of background genetic and environmental variables, including the microbiome.

There is abundant evidence of an immunological origin for IBD, influenced by genetic and environmental factors. Our results draw attention to a possible contribution from anti-IL-10 autoantibody-mediated disruption of immune regulation in some individuals. This event does not appear to be part of a general autoimmune diathesis (anti-IL-10 being absent from individuals we tested with autoimmune polyendocrinopathy-candidiasis-ectodermal-dystrophy (APECED), for example), but conceivably an environmental exposure such as EBV or CMV may provoke a cross-reactive humoral response in susceptible individuals^17^. We speculate that human anti-IL-10 plays a primary pathogenic role in IBD, but cannot exclude the possibility that autoantibody arises in the context of pre-existing inflammation. In that case, the anti-IL-10 autoantibodies might be an epiphenomenon or at most a disease-modifier. Non-neutralizing anti-IL-10 autoantibodies, previously described in certain patients with IBD and lupus erythematosus, presumably have a different functional significance with respect to IL-10 signalling and appear unlikely to influence gut inflammation^16 18^.

Notably, transcriptomic studies have identified a signature of IL-10 non-response in children with IBD^19^. Further studies are now needed to assess the prevalence of anti-IL-10 in IBD across the life course and its relationship to diverse disease phenotypes, (for example age at onset, disease progression, disease activity measures, surgical history, extra-intestinal manifestations, and response to therapy). Together with earlier reports of anti-GM-CSF autoantibodies as a marker of severity in IBD^8^ and anti-integrin αvβ6 autoantibodies in advance of UC development^20^, our findings highlight the potential contribution of humoral autoimmunity to the multifactorial pathogenesis of IBD and raise the possibility of humorally targeted therapy in selected individuals.

Disclosure forms provided by the authors are available with the full text of this article at NEJM.org.

## Supplementary Material

Supplement
