# Supplementary material for: Neutralizing Autoantibodies Against IL-10 in Inflammatory Bowel Disease
Source: N Engl J Med. Author manuscript; Available in PMC 2024 Aug 14. (PMC7616361; doi:10.1056/NEJMoa2312302)
Supplement: Supplement [file EMS195587-supplement-Supplement.pdf]

Neutralizing autoantibodies against IL-10 in inflammatory bowel disease

Helen Griffin*, Lourdes Ceron-Gutierrez*, Nima Gharahdaghi*, Soraya Ebrahimi, Sophie Davies, Peh Sun Loo, Andras Szabo, Eleri Williams, Anirban Mukhopadhyay, Louise McLoughlin, Steven Irwin, Simon Travis, Paul Klenerman, Su Bunn, Andrew J Cant, Sophie Hambleton[§¶], Holm H. Uhlig[§], Rainer Doffinger[§]

Supplementary appendix

Table of contents

Materials and methods	p2
Figure S1	p6
Figure S2	p7
Table S1	p9
Supplementary references	p11

Materials and methods

Samples & consent

Parents of the index cases provided written informed consent to publication of this report. Written informed consent was received from all controls participating in a REC-approved cohort study (Oxford IBD cohort study, REC 21/YH/0206). All data were anonymized, in accordance with protocols approved by the participating institutions. Healthy adult sera were salvaged from clinical laboratory IL-10 diagnostics, in which volunteer members of staff or patient relatives without known underlying disease are analyzed as controls. Such samples were anonymized other than gender and age.

Genetic screening

Exome sequences of P1 and parents were generated with Nextera Rapid Capture Kit (Illumina) and sequenced on NextSeq platform (Illumina). Sequences were aligned with BWA, duplicates marked by Picard, variants (SNVs, short indels) called with GATK and annotated with Ensembl VEP. Copy number variants were called with ExomeDepth. Variants with minor allele frequency <0.001 and predicted to alter protein sequence or disrupt splicing in genes associated with IBD or with a known immune function were assessed for potential pathogenicity. P2 underwent targeted next generation screening through NHS England.

Whole Blood assay.

Patient and control whole blood samples were pipetted into a sterile 96 well plate (Greiner Bio One Ltd, Cat No 651180). Bloods with medium alone (RPMI 1640, Gibco® #22400-089) were used as unstimulated controls. Stimulations were done using Lipopolysaccharide (LPS) (Biological Laboratories # 304) at final concentration of 2 µg/mL, alone or together with recombinant human Interleukin 10 (rhIL-10) (Immunotools #11340103) at a final concentration of 40 ng/mL. The final dilution for whole blood was 1:5. Plate was incubated for 24hr at 37°C and 5% CO_2. After incubation, supernatants were taken and kept frozen at -80°C until level of induced Tumor Necrosis Factor alpha (TNF-α) was measured.

Measurement of Tumor Necrosis Factor alpha (TNF-α).

Levels of TNF-α in supernatants were measured using a Human Magnetic Luminex Assay kit (R&D systems #LXSAHM) according to the manufacturer's instructions. Briefly, standards and

sample were added to a 96 well plate, followed by the microspheres; the plate was then incubated for 2 hours at room temperature on an orbital microplate shaker set at 700 rpm. After the incubation, the beads were washed three times with wash buffer and plate was incubated with biotinylated anti-TNF-α antibody for 60 minutes at room temperature on the orbital microplate shaker as before. Beads were washed and 50 µL of Streptavidin-PE (SA-PE) were added to each well and plate was incubated for 30 minutes at room temperature on orbital microplate shaker as before. The beads were then washed and re-suspended in wash buffer. TNF-α was measured with a Luminex 200 analyser (Bio-Plex, Bio-Rad Laboratories), according to the manufacturer's recommendations using Bio-Plex Manager 6.1 software for statistical analysis.

Anti-IL-10 serology

Recombinant human IL10 (rhIL-10) was covalently coupled to magnetic beads (Luminex, Netherlands). Beads were first activated with 1-ethyl-3-[3-dimethylaminopropyl]carbodiimide hydrochloride (Pierce / Thermo Fisher Scientific, #11851335) in the presence of N-hydroxysuccinimide (Pierce / Thermo Fisher Scientific, #10391314), according to the manufacturer's instructions, to form amine-reactive intermediates. The activated beads were then incubated with IL-10 at a concentration of 20 µg/mL in the reaction mixture for 3 h at room temperature on a rotator. Beads were washed and stored in blocking buffer (10 mM PBS, 1% BSA, 0.05% NaN_3). Successful coupling of the cytokine to its bead set was verified with specific mAb (R&D Systems, #BAF217).

IL10-coupled beads were incubated with patient sera for 1 h in 96-well F plates (Greiner Bio One #655096.) at room temperature in the dark on an orbital shaker. Plate was placed on a magnet and buffers were aspirated. Beads were washed three times with 10 mM PBS/0.05% Tween 20. Beads were then incubated for 30 min with a PE-labeled anti–human IgG-Fc antibody (Leinco/Biotrend; #I-127), washed as described, and resuspended in 100 µl PBS/Tween. They were then analysed on a Luminex analyser as described above.

IL-10 reporter assay (HEK-blue ™ cells)

The HEK-blue™ IL-10 cell line (InvivoGen # hkb-il10) can specifically respond to human IL10 by secreting alkaline phosphatase which can be measured colorimetrically at OD 620. Cells were cultured in growth medium, prepared with Dulbecco Modified Eagle Medium (DMEM,

Gibco®, # 10313021) supplemented with 10% fetal bovine serum (FBS, Sigma # F2442), 2mM L-Glutamine (Gibco ® # 25030081), 100 units/mL Penicillin, 100µg/mL Streptomycin (Gibco® # 15140122) and 100µg/mL of Normocin ™ (InvivoGen # ant-nr-1) for two passages. After a second passage the growth medium was replaced with selection medium plus HEK-blue ™selection antibiotics (InvivoGen hb-sel). Following 48 hrs in selection medium, cells were harvested and washed once with growth medium without Normocin™. The cells were re-suspended in growth medium without Normocin™, counted, and their viability assessed. Using a 96 well, flat bottom, sterile plate (CytoOne # CC7682-7596), harvested cells were stimulated with 4 ng/mL recombinant human IL-10 (ImmunoTools # 11340103) alone or in the presence of Control Sera (1:10) or sera of P1 and P2 (1:10). After 24 hrs of incubation, using an ELISA plate, 20 uL of each supernatant were added to 180 uL of QUANTI_Blue™ solution (InvivoGen # rep-qbs). The reactions were incubated for 60 minutes at 37˚C. Following the incubation, the optical density at 620 nm was measured to determine SEAP (secreted embryonic alkaline phosphatase) levels.

STAT3 dual luciferase reporter assay (HEK293 cells)

HEK293 cells, which naturally lack *IL10RA* expression, were cultured at a density of 30 × 10^3 cells per well in 96-well plate using DMEM (Sigma-Aldrich, #D6429). The medium was supplemented with 10% FBS (Sigma-Aldrich, #F9665), 1% penicillin (100 IU·mL^{-1}), and streptomycin (100 µg·mL^{-1}) (Sigma-Aldrich, #P0781). Cells were co-transfected with a vector encoding wild-type IL10RA (100 ng) and a STAT3 reporter (10 ng) (CCS-9028L, Cignal Reporter Assay Kit, #336841, QIAGEN) using Lipofectamine™ 3000 Transfection Reagent (#L3000001) in OPTI-MEM (reduced serum medium, Gibco, #31985-062). After 24-hours, co-transfected cells were exposed to recombinant human IL-10 (Peprotech, #200-10) 200 ng·mL^{-1}, in the presence or absence of control sera (1:10) or sera of P1 or P2 (1:10). Eventually, luciferase activity was assessed using the Dual-Luciferase® Reporter Assay System (Promega, E1960), and measurements were performed as per the manufacturer's instructions.

IL-10 cross inhibition assay.

PBMCs from healthy donor and patient were isolated using Ficoll-Hypaque (Axis-Shield Lymphoprep #1114545) density gradient centrifugation. Isolated PBMCs were washed twice with sterile medium RPMI 1640 and adjusted to 5X10^6 viable cells per mL. In a sterile cell

culture plate (Greiner Bio One Ltd, #651180), 20 µL of serum from patient and healthy control were added to the corresponding wells. Stimulants were added as follows: a) Medium (RPMI 1640, Gibco® #22400-089) as unstimulated control; b) LPS to final concentration of 2 µg/mL; c) rhIL-10 to final concentration of 40 ng/mL or d) LPS plus rhIL-10 to obtain a final volume of 80 µL / well. The plate was put for 2 minutes on an orbital shaker and incubated at room temperature for 30 minutes. Following the incubation, 20 µL of each cell suspension ($1x10^5$ cells per well) were added to each condition. Plate was then incubated for 24hr at 37°C and 5% CO2. Supernatants were taken and kept frozen at -80°C until level of induced TNF-α was measured.

Figure S1: Serial immunoglobulin measurements in P1 serum over time

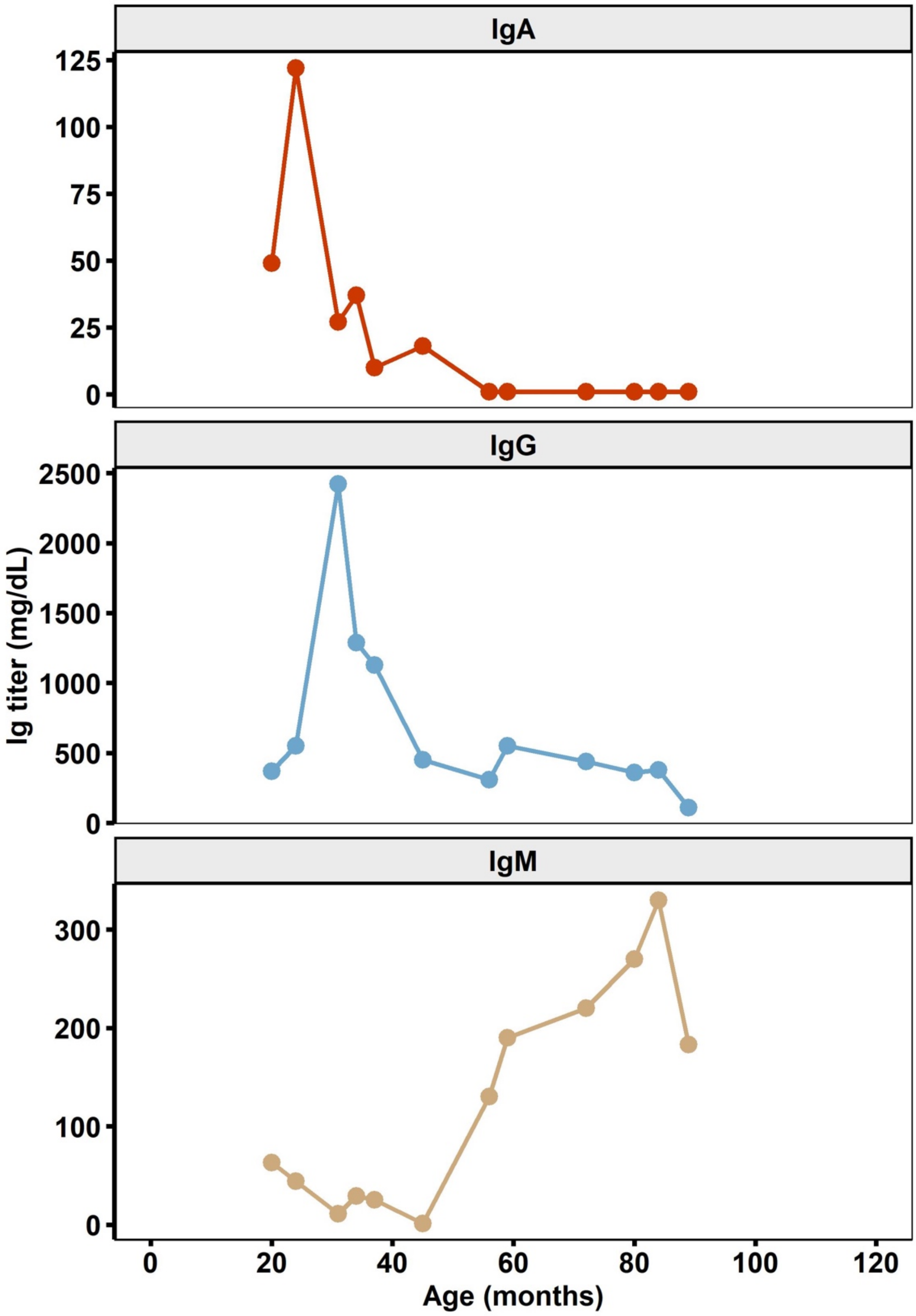

Figure S2

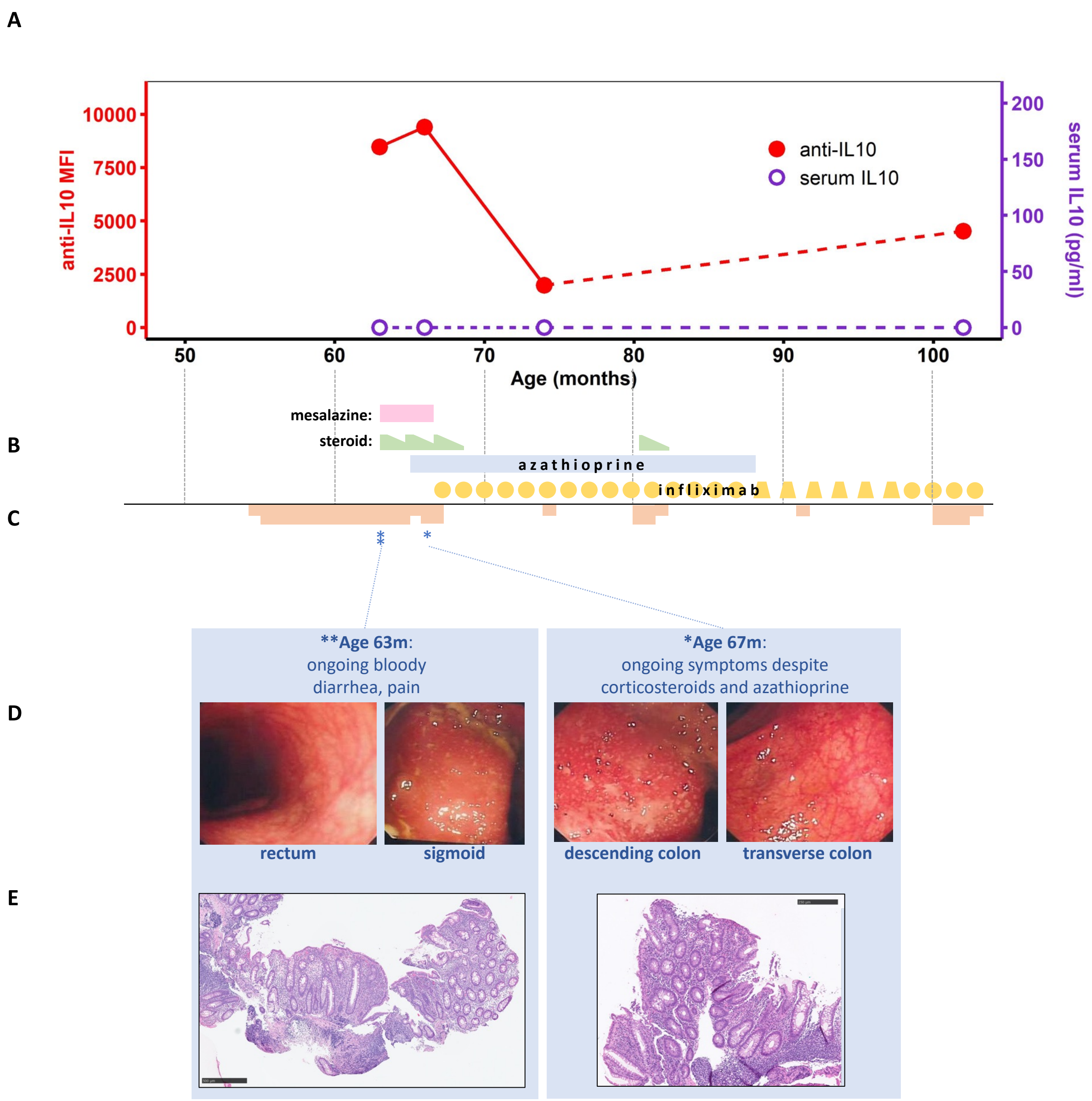

Clinical and laboratory timeline of very early onset inflammatory bowel disease (VEOIBD) in P2, a child with a raised titer of neutralizing autoantibodies to interleukin-10.

(A) Anti-IL-10 titer (red, Mean Fluorescence Intensity (MFI) by particle-based assay, 1:100) and serum IL-10 (purple) measured over time while receiving therapy as indicated in (B). Infliximab was given at a dose of 10 mg/kg 6-weekly except between months 88 and 100 when it was given 8-weekly. (C) Clinical timeline in which the severity of GI symptoms (correlating with fecal calprotectin) is indicated by the height of the pink shading. Asterisks show the timing of endoscopic examinations. (D) Colonoscopic appearances at 63 and 67 months,

illustrating failure to remit on weaning corticosteroid despite azathioprine therapy. Endoscopic findings: significant colitis from rectum to 60 cm (mid transverse) in continuous fashion with loss of vascular pattern and multiple small aphthous ulcers. Then normal mucosa up until cecum - aphthous ulcers seen in cecum (not shown). Terminal ileum normal. Normal peri-anal appearances. Mild gastritis. (E), representative images of colonic histopathology, stained with haematoxylin and eosin. Left, (63m): chronic mucosal damage with crypt bifurcation and distortion. Mild active colitis. Scale-bar 500µm. Right, (67m): mild active pan-colitis with only subtle evidence of chronicity. No granulomas. Scale-bar, 250µm

Table S1: Summary of prior literature reporting anti-IL-10 autoantibodies in healthy controls or a variety of disease states

Reference	Study population (Disease / control)	Age (y)	Positive anti-IL10 among disease group	Positive anti-IL10 among controls	Anti-IL10 association with disease?	Neutralization of IL-10 reported (method)?	Causal relationship between antibody and disease?
Menetrier-Caux, et al (1996) [1]	Various autoimmune, diseases, lymphoma, malaria, IgA deficiencies and enterocolitis	Adults	Chronic inflammatory arthritis: 3/400 (ns) Pemphigus/pemphigoid: 4/300 (ns) Myasthenia gravis: 0/100 Lupus erythematosus: 0/45 Lymphoma: 0/58 Malaria: 0/130 Crohn's disease/enterocolitis: 0/42 IgA deficiencies: 0/16	0/133	Chronic inflammatory arthritis and Pemphigus/ pemphigoid	YES (Inhibition of IL-10-dependent growth of cell line by affinity-purified autoantibodies)	Statistically unclear
Tanner, et al (1997) [2]	EBV infectious mononucleosis	Adults	lymphoproliferative disease: 6/10 chronic infectious mononucleosis: 2/5 nasopharyngeal carcinoma: 2/5 EBV-positive: 0/10 acute infectious mononucleosis: 0/10 healthy transplant: 0/10	0/10	CIM, NPC, and LPD	YES (Inhibition of IL-10-dependent growth of cell line)	Anti–IL-10 antibodies in 1 CIM patient could neutralize IL-10 bioactivity in vitro
Watanabe, et al (2007) [3]	Healthy control individuals	Male: 30-51 (n=5) Female: 20-49 (n=6) +3 plasma +1 IVIG samples	No disease model in this study	11/15		NO	N/A

Ebert, et al (2009) [4]	Ulcerative colitis and Crohn's disease	UC: 43±10 (n=136)<br>CD: 45±8 (n=81)<br>Control: 44±8 (n=58)	UC: 15/136<br>CD: 14/81<br>Highest control values are considered as cut-off	0/58	IBD	*YES (3-(4,5-dimethylthiazol-2-yl)-2,5-diphenyltetrazolium bromide (MTT) assay vs CTLL cells)	*Relationship between ELISA positivity and neutralizing activity weak.<br>Rise in anti-cytokine antibodies was not found in a particular patient subset.
Frede, et al (2014) [5]	Ulcerative colitis and Crohn's disease	Adults	UC: 1/14<br>CD: 7/38<br>Highest control values are considered as cut-off	0/20	Crohn's disease	NO (STAT3 phosphorylation ELISA assay using PBMC)	No neutralizing activity demonstrated by STAT3 phosphorylation assay
Uchida, et al (2019) [6]	Systemic lupus erythematosus	44.7±13.8	systemic lupus erythematosus: 14/80<br>Systemic Scleroderma: 1/16<br>Rheumatoid Arthritis: 1/19<br>Behcet's disease: 0/23<br>Highest control values are considered as cut-off	0/23	Systemic lupus erythematosus, Rheumatoid Arthritis, Systemic Sclerosis	NO (None performed)	N/A

References

1. Menetrier-Caux C, Briere F, Jouvenne P, Peyron E, Peyron F, Banchereau J. Identification of human IgG autoantibodies specific for IL-10. Clin Exp Immunol 1996;104(1):173-9. DOI: 10.1046/j.1365-2249.1996.d01-646.x.
2. Tanner JE, Diaz-Mitoma F, Rooney CM, Alfieri C. Anti-interleukin-10 antibodies in patients with chronic active Epstein-Barr virus infection. J Infect Dis 1997;176(6):1454-61. DOI: 10.1086/514141.
3. Watanabe M, Uchida K, Nakagaki K, et al. Anti-cytokine autoantibodies are ubiquitous in healthy individuals. FEBS Lett 2007;581(10):2017-21. DOI: 10.1016/j.febslet.2007.04.029.
4. Ebert EC, Panja A, Das KM, et al. Patients with inflammatory bowel disease may have a transforming growth factor-beta-, interleukin (IL)-2- or IL-10-deficient state induced by intrinsic neutralizing antibodies. Clin Exp Immunol 2009;155(1):65-71. DOI: 10.1111/j.1365-2249.2008.03802.x.
5. Frede N, Glocker E-O, Wanders J, et al. Evidence for non-neutralizing autoantibodies against IL-10 signalling components in patients with inflammatory bowel disease. BMC immunology 2014;15:1-6.
6. Uchida M, Ooka S, Goto Y, et al. Anti-IL-10 antibody in systemic lupus erythematosus. Open Access Rheumatology: Research and Reviews 2019:61-65.
